# Supplementary material for: Suit the Remedy to the Case—The Effectiveness of COVID-19 Nonpharmaceutical Prevention and Control Policies Based on Individual Going-Out Behavior
Source: Int J Environ Res Public Health. 2022 Dec 4;19(23):16222. doi: 10.3390/ijerph192316222 (PMC9739683; doi:10.3390/ijerph192316222)
Supplement: Supplementary file 1 [file ijerph-19-16222-s001.zip › ijerph-2006539-supplementary.pdf]

Table S1. Peak value of infections, peak arrival time, epidemic duration and infection curve flatness index corresponding to all experimental scenarios.

Experimental scenario	Peak value of infections	Peak arrival time	Epidemic duration	Flatness index of infection curve
Experiment 1-1	1944.56	21	48	0.0247
Experiment 1-2	1259.68	45	119	0.0945
Experiment 1-3	119.42	152	428	3.5840
Experiment 2-1	1683.72	31	77	0.0457
Experiment 2-2	1379.06	40	105	0.0761
Experiment 2-3	1840.24	25	63	0.0342
Experiment 2-4	1565.86	34	91	0.0581
Experiment 2-5	1892.28	23	54	0.0285
Experiment 2-6	1761.84	29	68	0.0386
Experiment 3-1	1161.96	48	114	0.0981
Experiment 3-2	485.44	87	218	0.4491
Experiment 3-3	889.84	60	137	0.1540
Experiment 3-4	593.68	79	186	0.3133
Experiment 3-5	548.48	78	228	0.4157
Experiment 3-6	356.78	103	245	0.6867
Experiment 4-1-1	105.04	177	422	4.0175
Experiment 4-1-2	106.56	155	499	4.6828
Experiment 4-1-3	151.58	139	329	2.5861
Experiment 4-2-1	1928.36	22	50	0.0259
Experiment 4-2-2	1928.54	22	49	0.0254
Experiment 4-2-3	1937.46	20	50	0.0258
Experiment 4-3-1	1255.86	47	102	0.0812
Experiment 4-3-2	1261.94	46	102	0.0808
Experiment 4-3-3	1282.26	42	104	0.0811

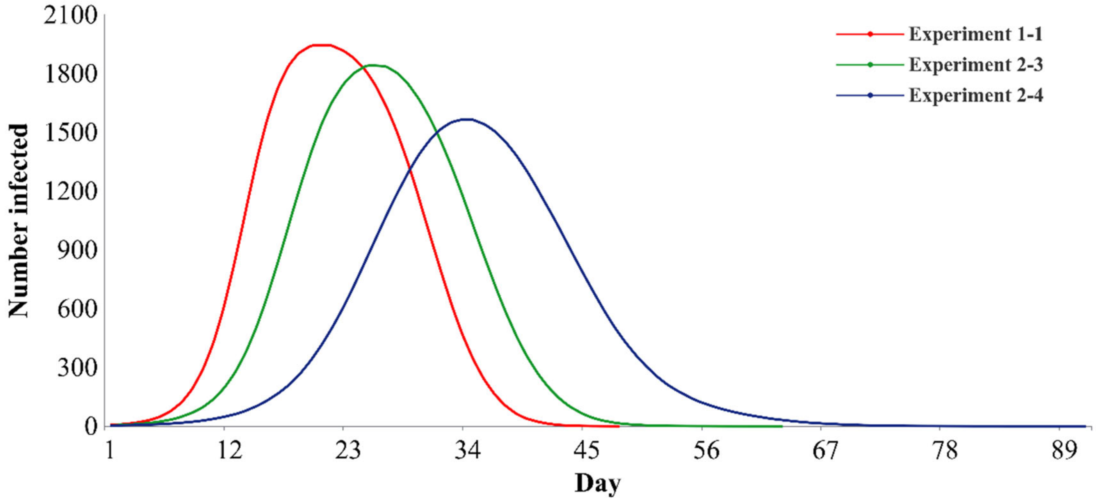

Figure S1. Infection curves of different intensities of the RCONT policy (the other policies are loose).

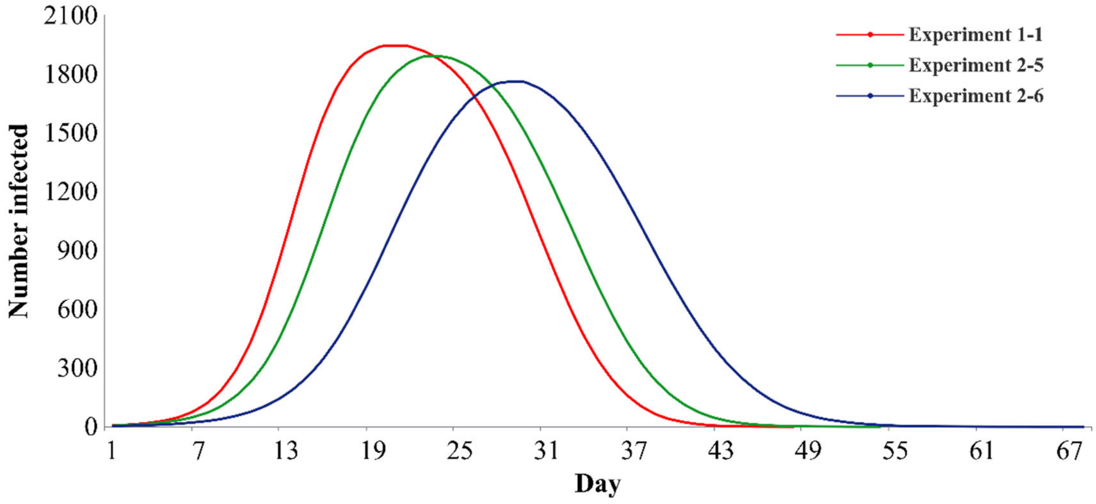

Figure S2. Infection curves of different intensities of the RINFE policy (the other policies are loose).

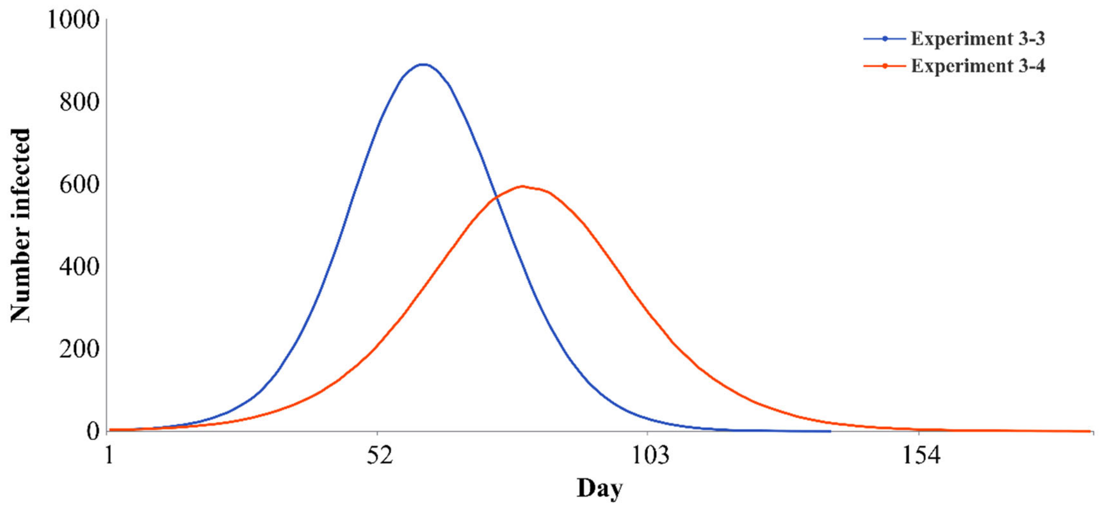

Figure S3. Infection curves of different intensities of the RCONT policy (the other policies are strict).

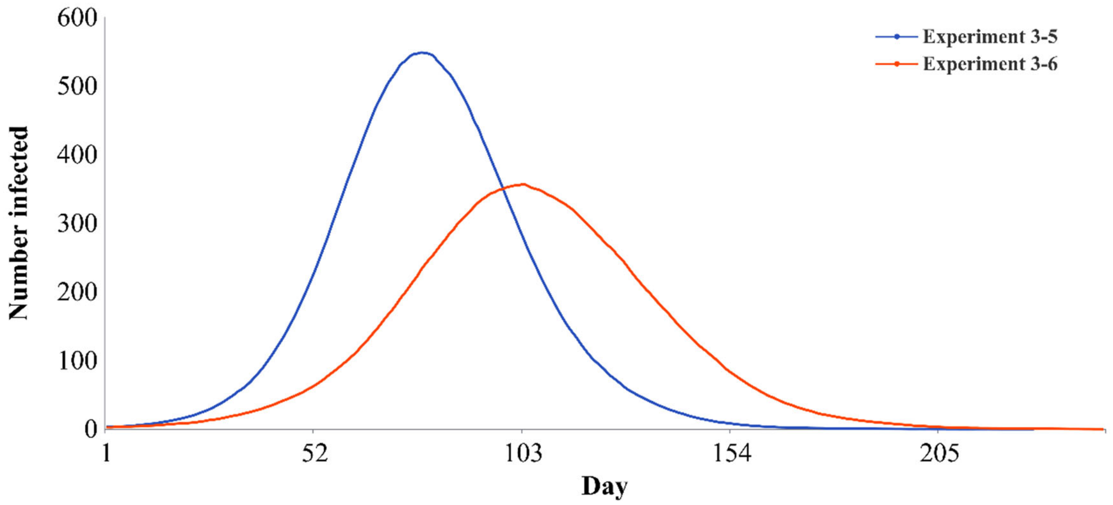

Figure S4. Infection curves of different intensities of the RCONT policy (the other policies are strict).

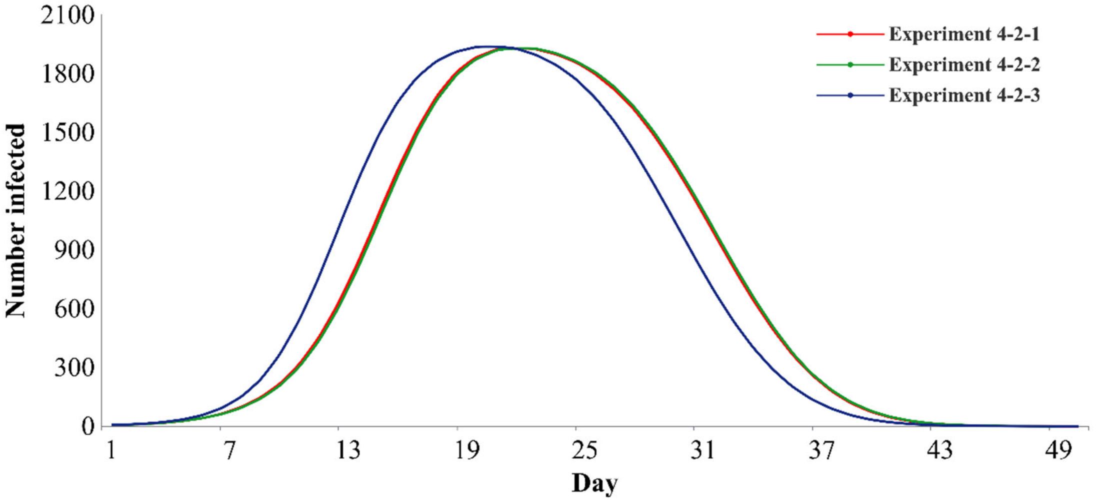

Figure S5. Infection curves of different kinds of going-out needs with loose policy intensity.

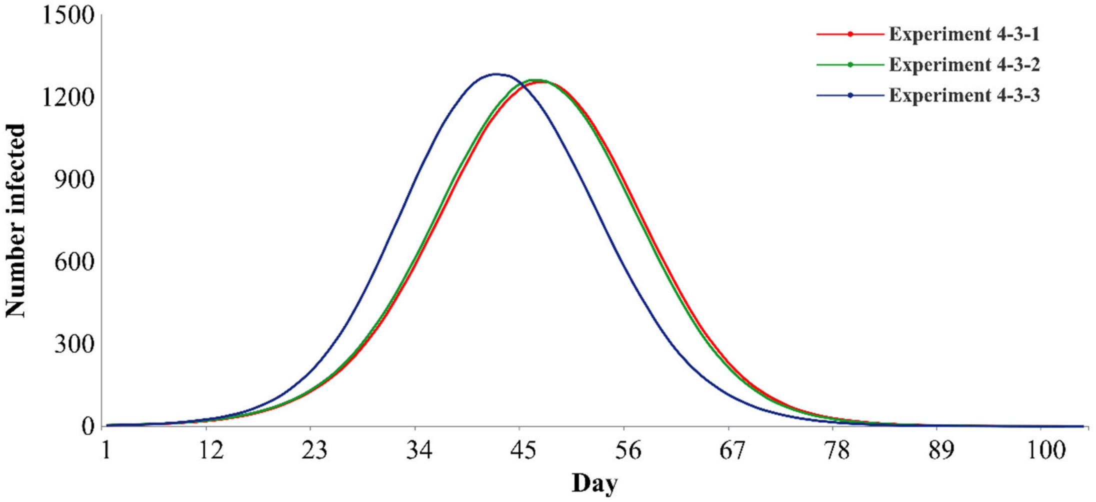

Figure S6. Infection curves of different kinds of going-out needs with moderate policy intensity.

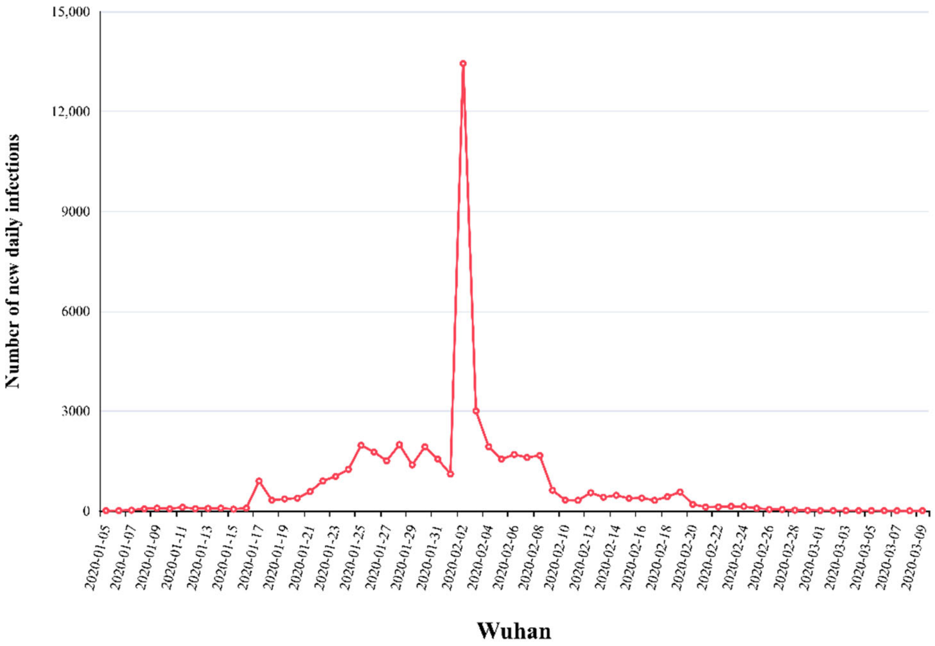

Figure S7. Curve of new daily infections in Wuhan, China (2020.01.05-2020.03.09). This paper selects data from new daily infections from January 5, 2020, to March 9, 2020, in Wuhan, China, which reached a peak of 13,436 on February 2, 2020. Compared with the results of basic experiments based on three going-out needs, Wuhan is comparatively in line with the scenario of Experiment 1-1.

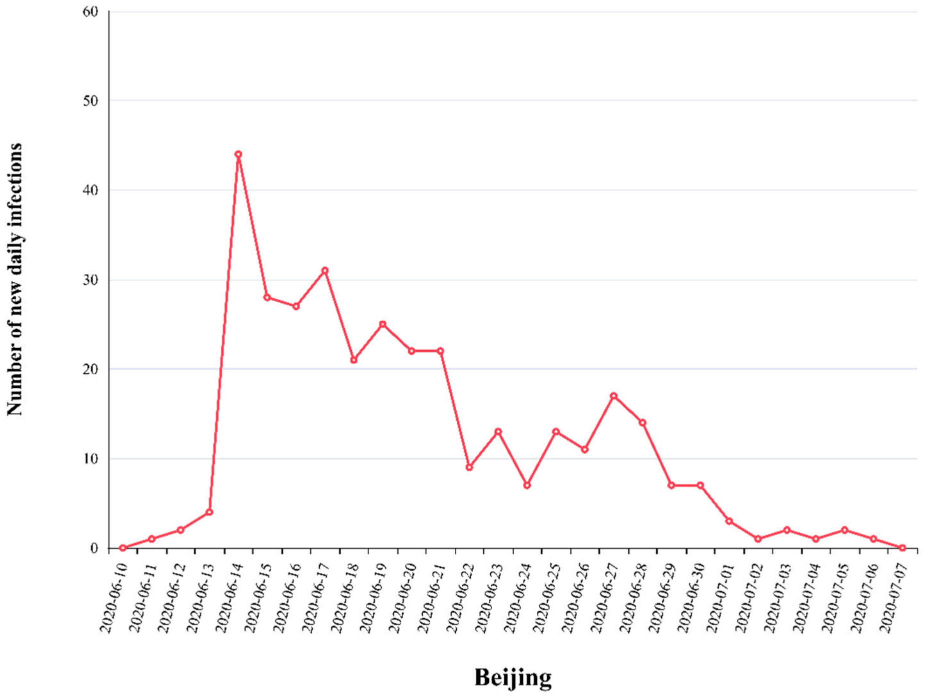

Figure S8. Curve of new daily infections in Beijing, China (2020.06.10-2020.07.07). This paper selects the data from new daily infections from June 10, 2020, to July 7, 2020 in Beijing, China,

which reached a peak of 44 on June 14, 2020. Compared with the results of basic experiments based on three going-out needs, Beijing is comparatively in line with the scenario of Experiment 1-2.

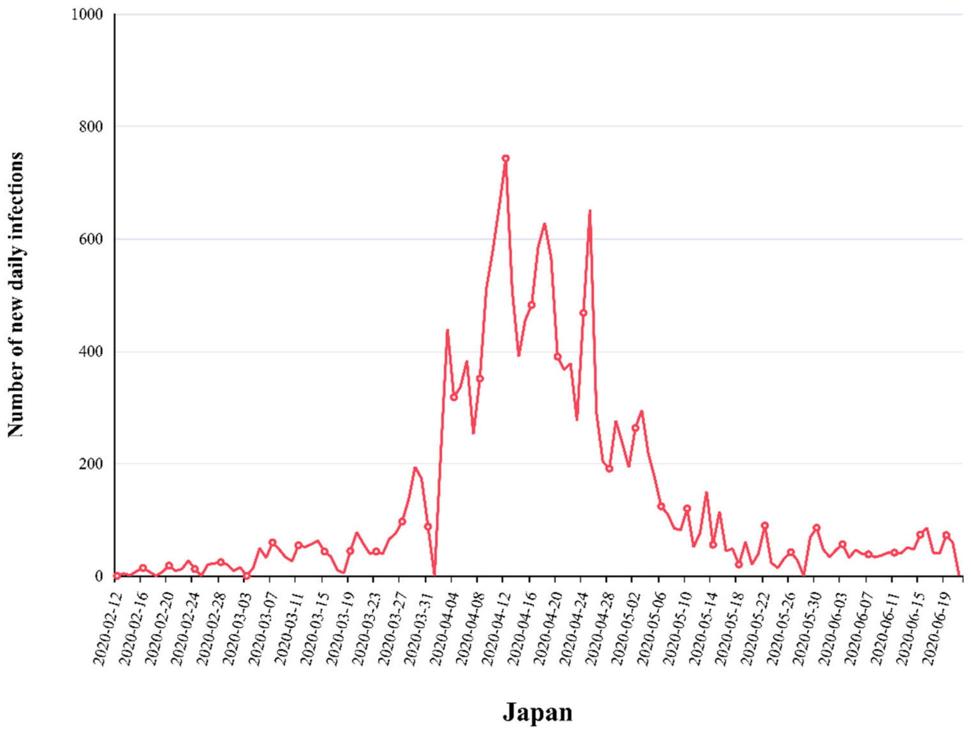

Figure S9. Curve of new daily infections in Japan (2020.02.12-2020.06.21). This paper selects the data from new daily infections from February 12, 2020, to June 21, 2020, in Japan, which reached a peak of 743 on April 12, 2020. Compared with the results of basic experiments based on three going-out needs, Japan is comparatively in line with the scenario of Experiment 1-2.

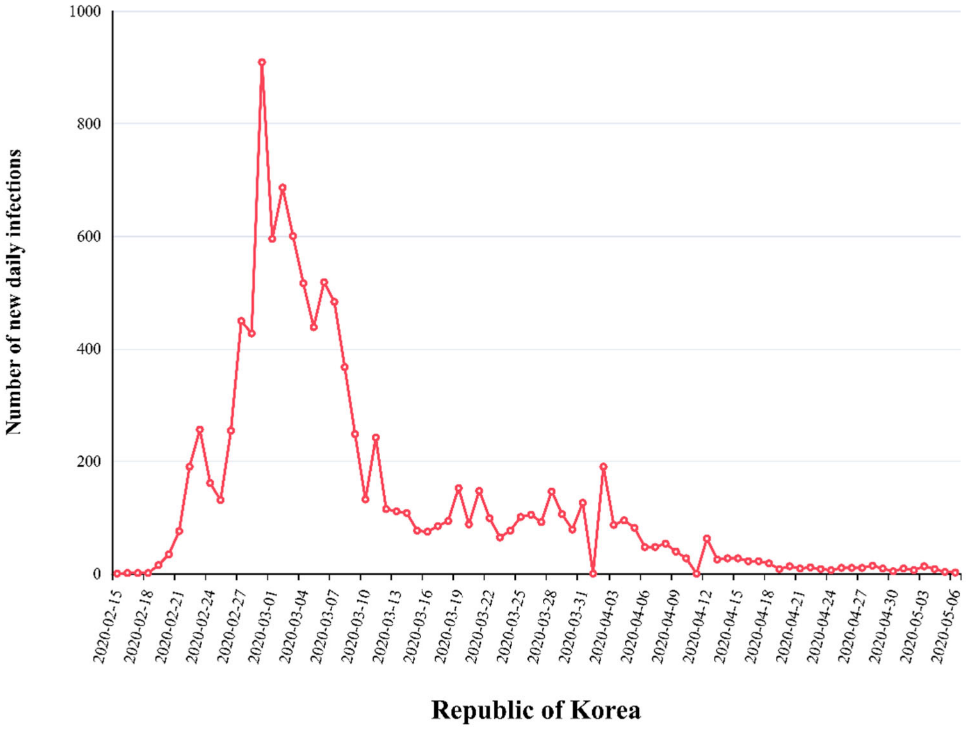

Figure S10. Curve of new daily infections in the Republic of Korea (2020.02.15-2020.05.06). This paper selects data from new daily infections from February 15, 2020, to May 6, 2020, in the Republic of Korea, which reached a peak of 909 on February 29, 2020. Compared with the results of basic experiments based on three going-out needs, the Republic of Korea is comparatively in line with the scenario of Experiment 1-1.

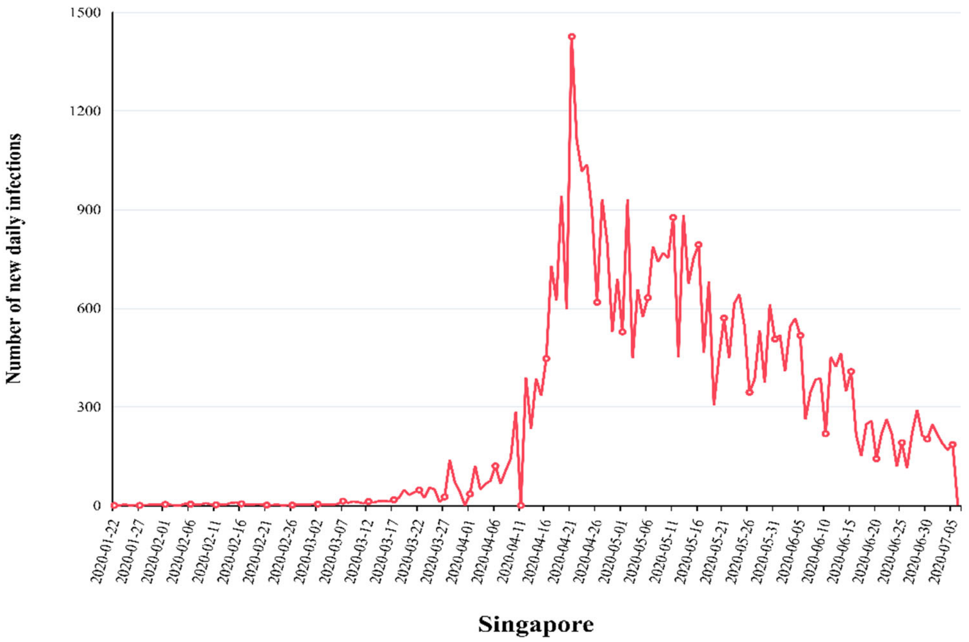

Figure S11. Curve of new daily infections in Singapore (2020.01.22-2020.07.06). This paper

selects data from new daily infections from January 22, 2020, to July 6, 2020 in Singapore, which reached a peak of 1426 on April 21, 2020. Compared with the results of basic experiments based on three going-out needs, Singapore is comparatively in line with the scenario of Experiment 1-2.

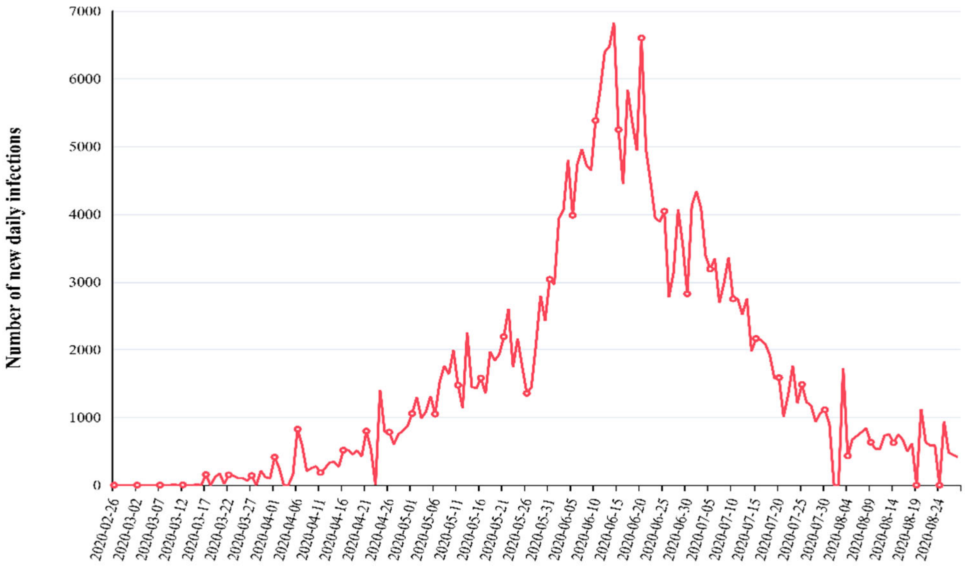

Pakistan

Figure S12. Curve of new daily infections in Pakistan (2020.02.26-2020.08.28). This paper selects data from new daily infections from February 26, 2020, to August 28, 2020, in Pakistan, which reached a peak of 6825 on June 14, 2020. Compared with the results of basic experiments based on three going-out needs, Pakistan is comparatively in line with the scenario of Experiment 1-2.

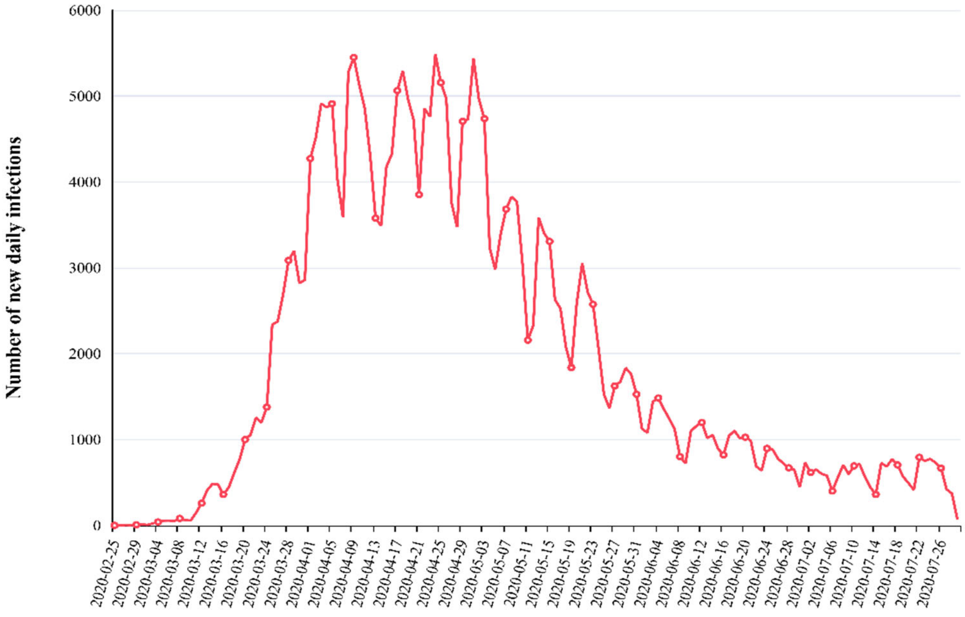

United Kingdom

Figure S13. Curve of new daily infections in the United Kingdom (2020.02.25-2020.07.29). This

paper selects the data from new daily infections from February 25, 2020, to July 29, 2020, in the United Kingdom, which reached a peak of 5487 on April 24, 2020. Compared with the results of basic experiments based on three going-out needs, the United Kingdom is comparatively in line with the scenario of Experiment 1-2.

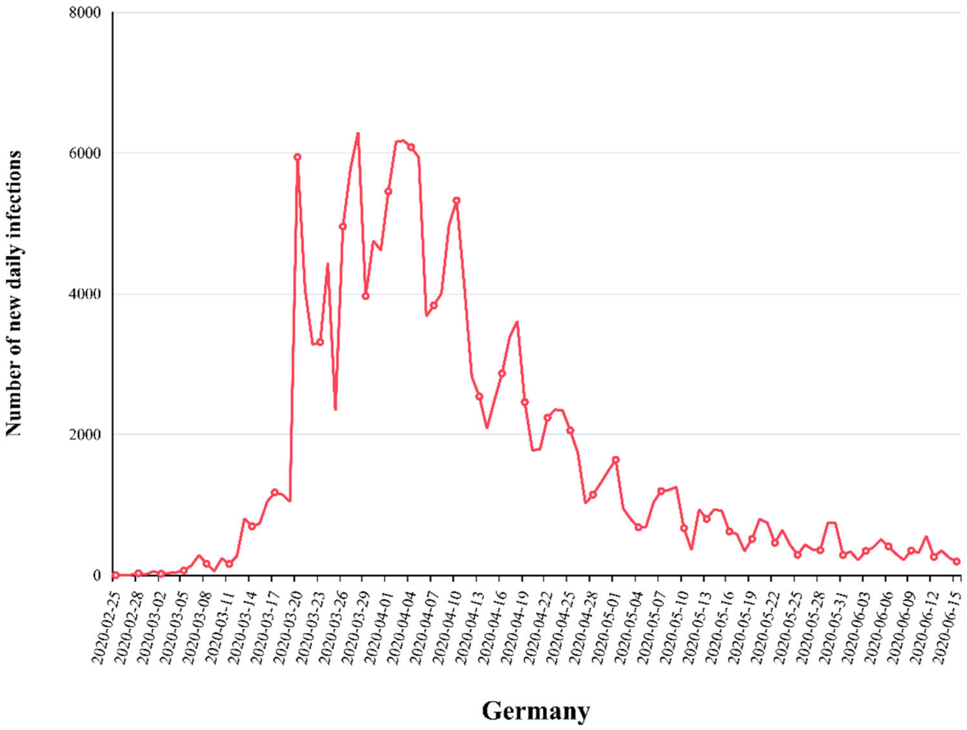

Figure S14. Curve of new daily infections in Germany (2020.02.25-2020.06.15). This paper selects the data from new daily infections from February 25, 2020, to June 15, 2020, in Germany, which reached peaks on March 20, March 28, and April 4, respectively. The numbers of new infections on these three days were 5940, 6294 and 6174 and showed a downward trend after April 4. Compared with the results of basic experiments based on three going-out needs, Germany is comparatively in line with the scenario of Experiment 1-1.

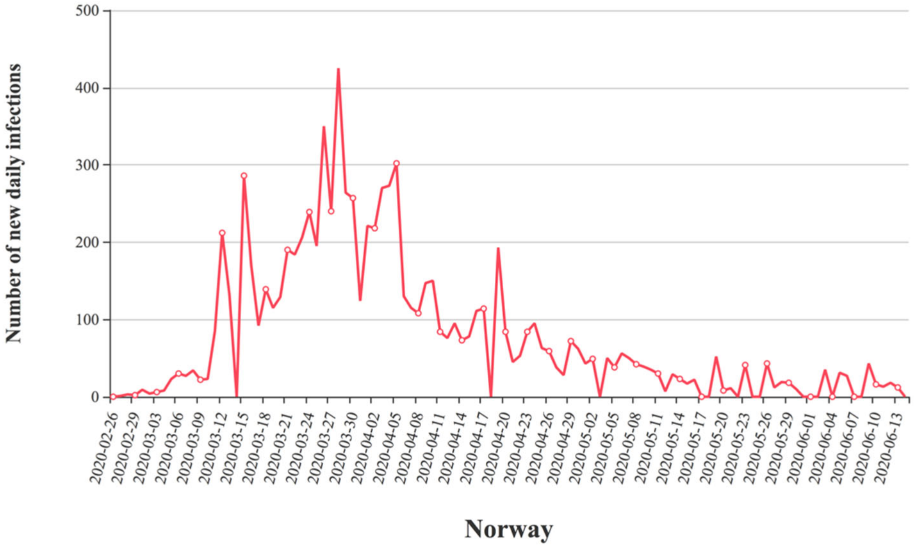

Figure S15. Curve of new daily infections in Norway (2020.02.26-2020.06.13). This paper selects the data from new daily infections from February 26, 2020, to June 13, 2020, in Norway, which reached a peak of 425 on March 28, 2020. Compared with the results of basic experiments based on three going-out needs, Norway is comparatively in line with the scenario of Experiment 1-1.

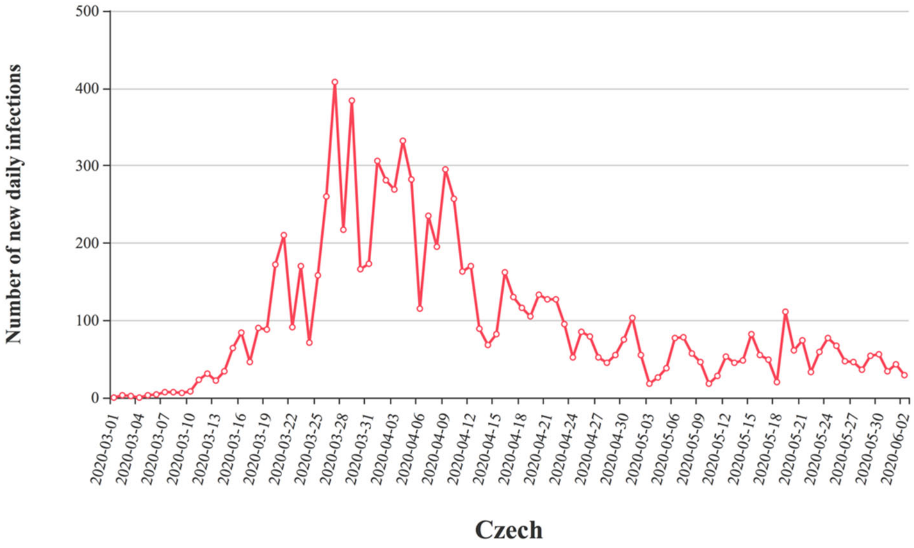

Figure S16. Curve of new daily infections in Czech (2020.03.01-2020.06.02). This paper selects from data of new daily infections from March 1, 2020, to June 2, 2020, in Czech, which reached a peak of 408 on March 27, 2020. Compared with the results of basic experiments based on three going-out needs, Czech is comparatively in line with the scenario of Experiment 1-3.

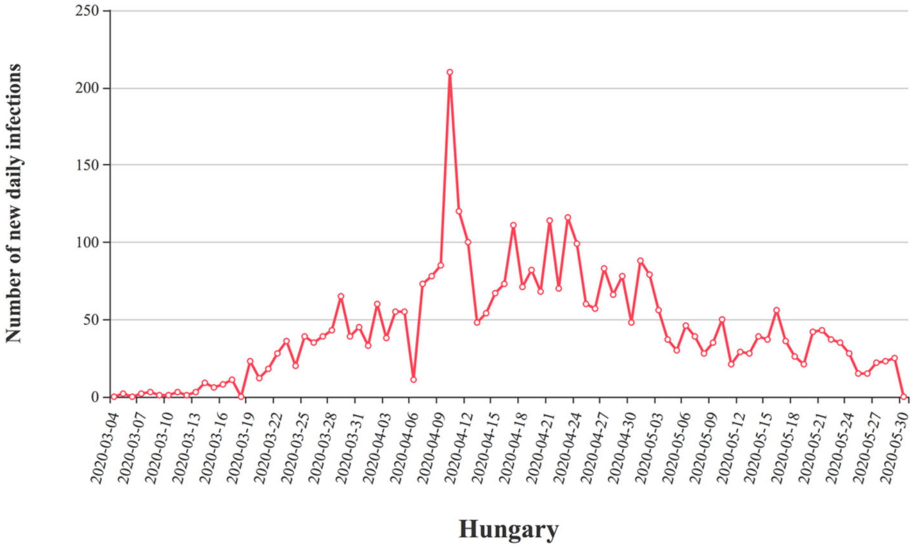

Figure S17. Curve of new daily infections in Hungary (2020.03.04-2020.05.30). This paper selects the data from new daily infections from March 4, 2020, to May 30, 2020, in Hungary, which reached a peak of 210 on April 10, 2020. Compared with the results of basic experiments based on three going-out needs, Hungary is comparatively in line with the scenario of Experiment 1-2.

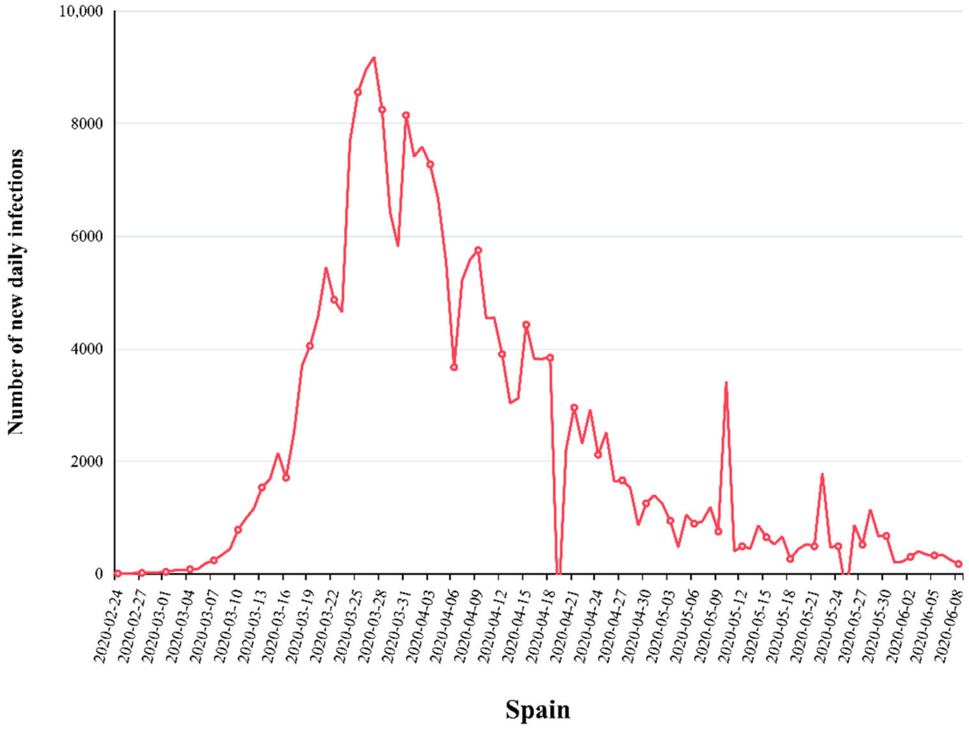

Figure S18. Curve of new daily infections in Spain (2020.02.24-2020.06.07). This paper selects data from new daily infections from February 24, 2020, to June 8, 2020, in Spain, which reached a

peak of 9181 on March 27, 2020. Compared with the results of basic experiments based on three going-out needs, Spain is comparatively in line with the scenario of Experiment 1-1.

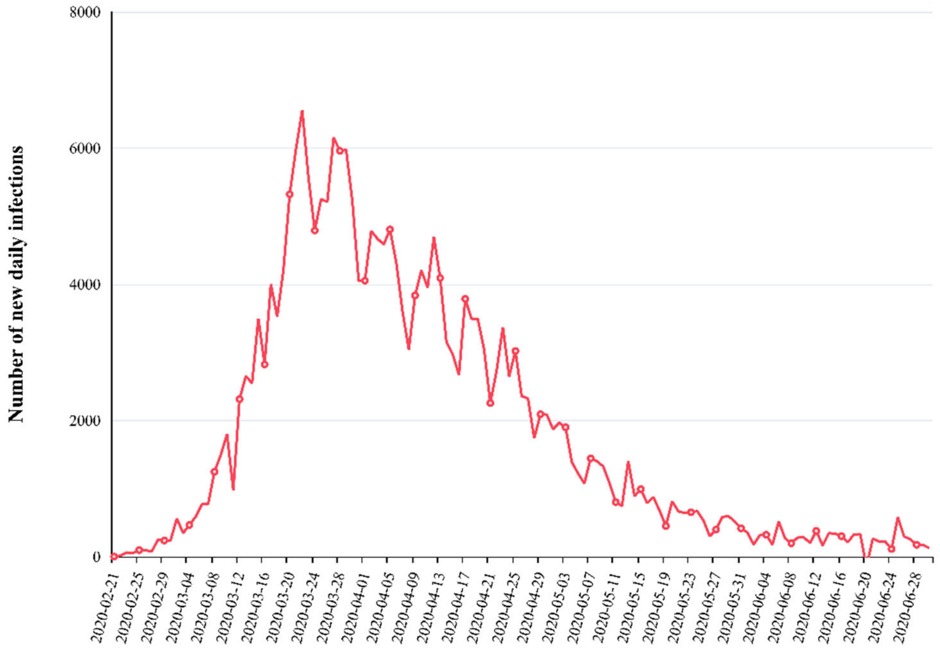

Italy

Figure S19. Curve of new daily infections in Italy (2020.02.21-2020.06.30). This paper selects data from new daily infections from February 21, 2020, to June 30, 2020, in Italy, which reached a peak of 6557 on March 22, 2020. Compared with the results of basic experiments based on three going-out needs, Italy is comparatively in line with the scenario of Experiment 1-2.

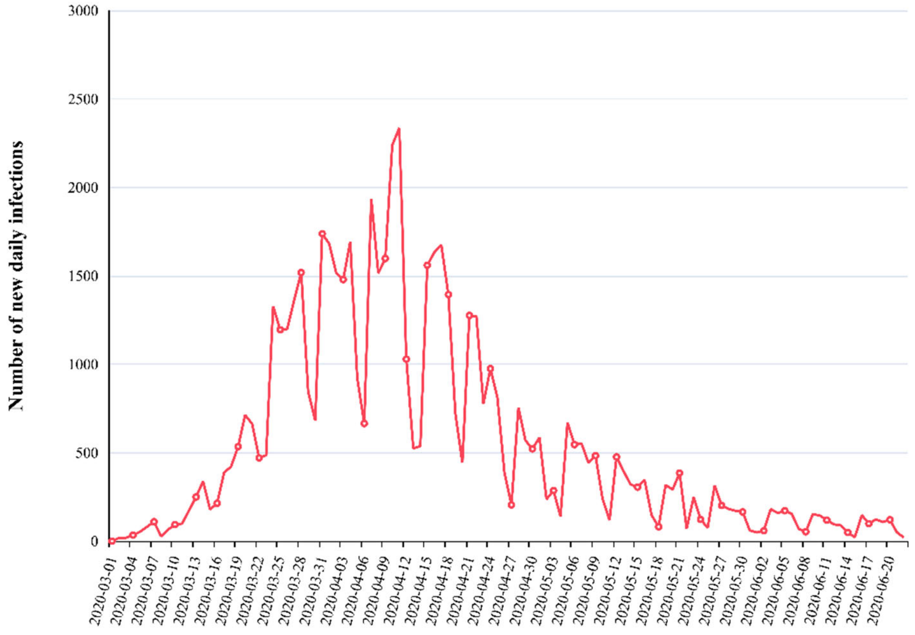

Belgium

Figure S20. Curve of new daily infections in Belgium (2020.03.01-2020.06.21). This paper selects data from new daily infections from March 1, 2020, to June 22, 2020, in Belgium, which reached a peak of 2336 on April 11, 2020. Compared with the results of basic experiments based on three going-out needs, Belgium is comparatively in line with the scenario of Experiment 1-3.

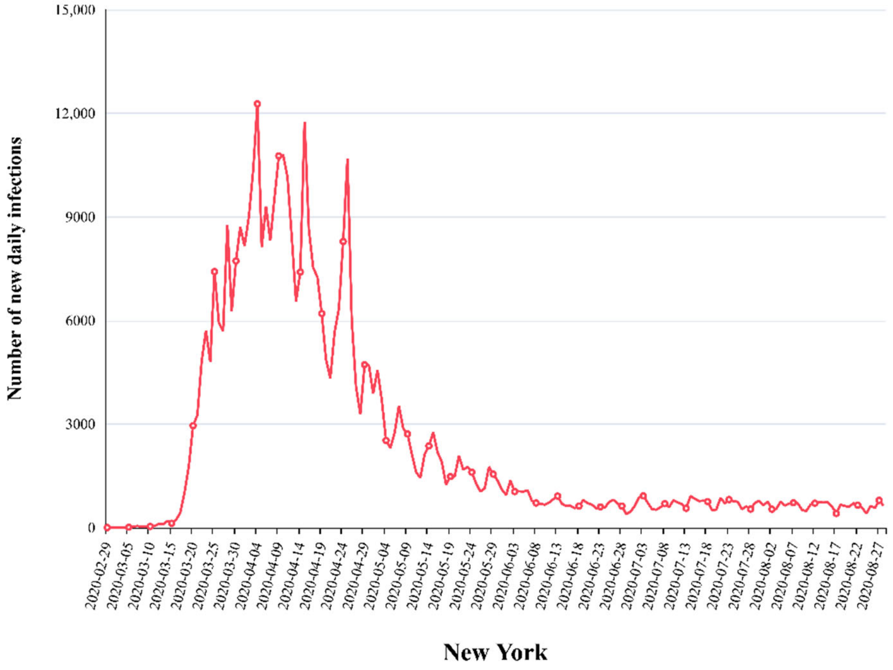

Figure S21. Curve of new daily infections in New York, USA (2020.02.29-2020.08.27). This paper selects the data from new daily infections from February 29, 2020, to August 28, 2020, in New York, which reached a peak of 12,247 on April 4, 2020. Compared with the results of basic experiments based on three going-out needs, New York is comparatively in line with the scenario of Experiment 1-2.

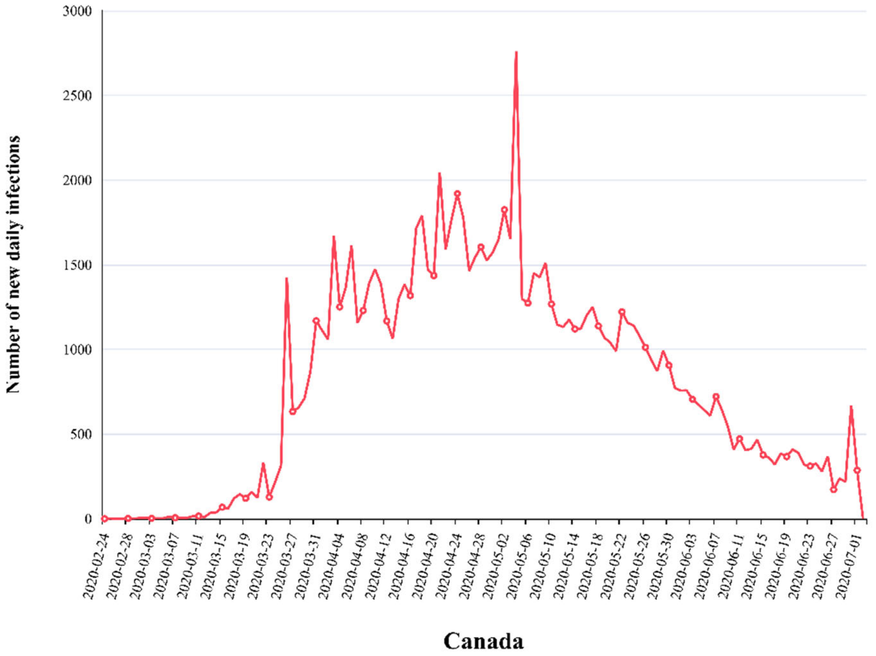

Figure S22. Curve of new daily infections in Canada (2020.02.24-2020.07.01). This paper selects data from new daily infections from February 24, 2020, to July 2, 2020, in Canada, which reached a peak of 2760 on May 4, 2020. Compared with the results of basic experiments based on three going-out needs, Canada is comparatively in line with the scenario of Experiment 1-2.

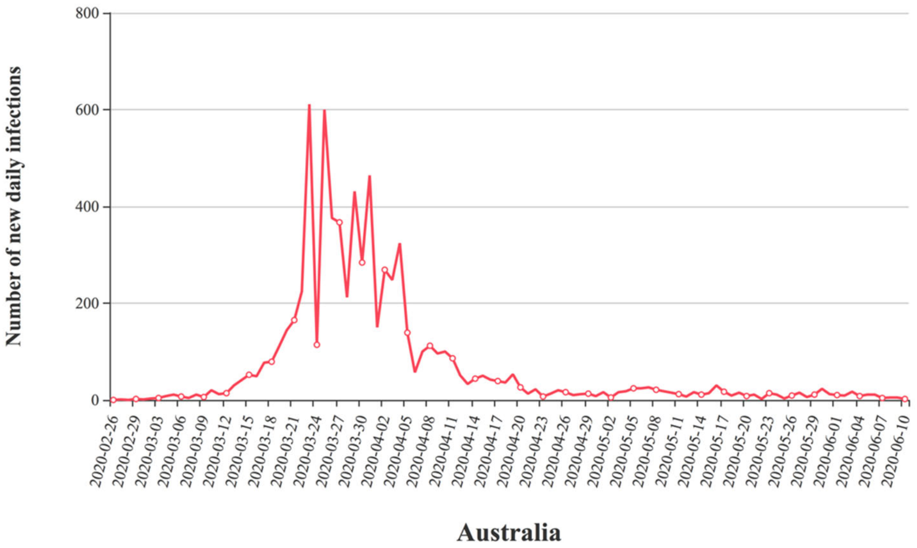

Figure S23. Curve of new daily infections in Australia (2020.02.26-2020.06.09). This paper selects the data from new daily infections from February 26, 2020, to June 10, 2020, in Australia, which reached a peak of 611 on March 23, 2020. Compared with the results of basic experiments based on three going-out needs, Australia is comparatively in line with the scenario of Experiment

1-1.

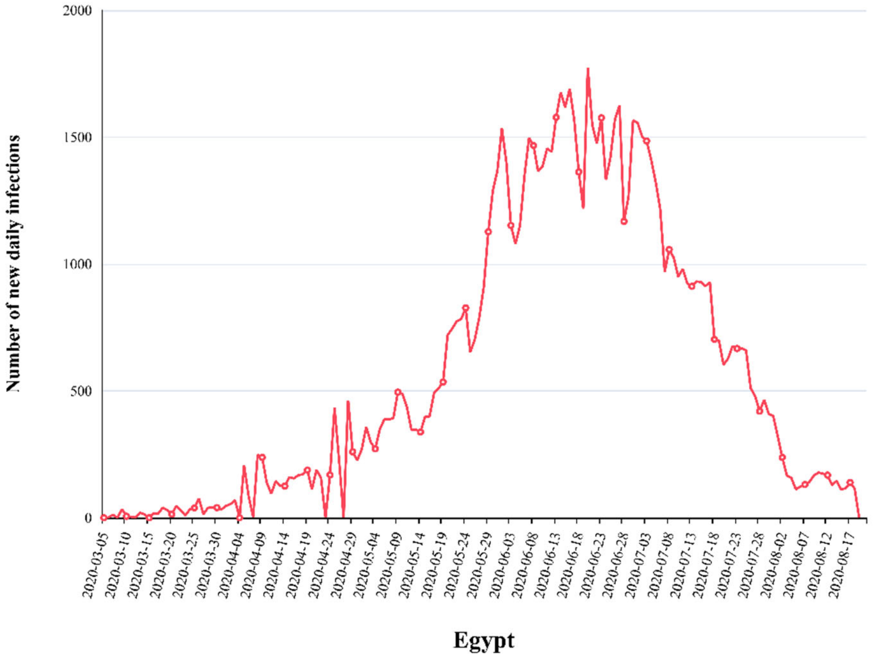

Figure S24. Curve of new daily infections in Egypt (2020.03.05-2020.08.19). This paper selects data from new daily infections from March 5, 2020, to August 19, 2020, in Egypt, which reached a peak of 1774 on June 20, 2020. Compared with the results of basic experiments based on three going-out needs, Egypt is comparatively in line with the scenario of Experiment 1-2.

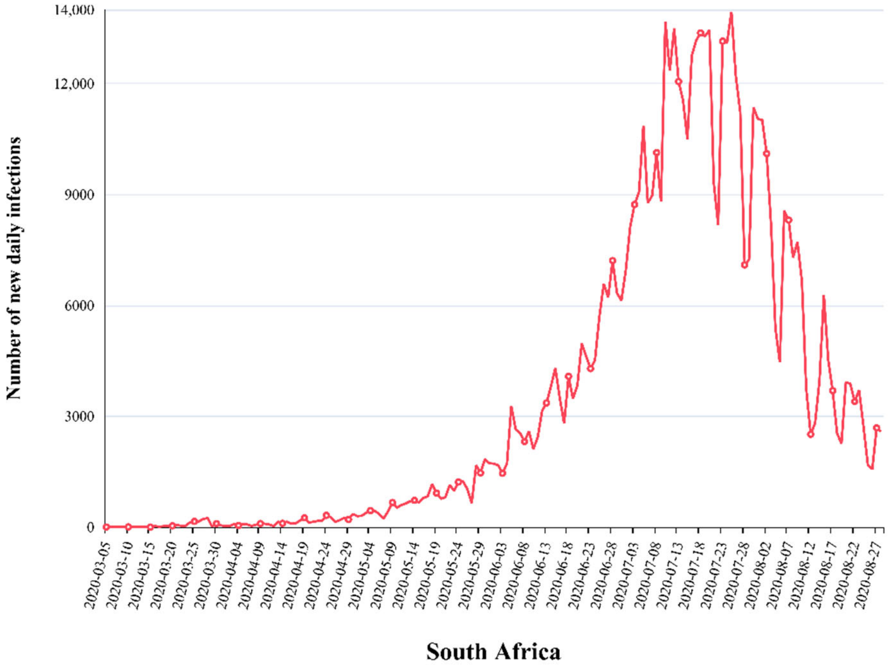

Figure S25. Curve of new daily infections in South Africa (2020.03.05-2020.08.28). This paper selects the data from new daily infections from March 5, 2020, to August 28, 2020, in South Africa,

which reached a peak of 13,944 on July 25, 2020. Compared with the results of basic experiments based on three going-out needs, South Africa is comparatively in line with the scenario of Experiment 1-2.
